# Examination of Stress Corrosion Cracking of Rock Bolts in Simulated Underground Environments

**DOI:** 10.3390/ma18061275

**Published:** 2025-03-13

**Authors:** Saisai Wu, Xinting Cao, Yiran Zhu, Krzysztof Skrzypkowski, Krzysztof Zagórski

**Affiliations:** 1 Shanxi Key Laboratory of Geotechnical and Underground Space Engineering, School of Resources Engineering, Xi’an University of Architecture and Technology (XAUAT), Xian 710055, China; 2 School of Mechanical & Mining Engineering, University of Queensland, St. Lucia, QLD 4072, Australia; 3 Faculty of Civil Engineering and Resource Management, AGH University of Krakow, Mickiewicza 30 Av., 30-059 Kraków, Poland; 4 Faculty of Mechanical Engineering and Robotics, AGH University of Krakow, Mickiewicza 30 Av., 30-059 Kraków, Poland

**Keywords:** premature failures, rock bolts, acidified solution, underground conditions, hydrogen-induced SCC

## Abstract

In recent years, significant increases in premature failures of rock bolts that are attributed to stress corrosion cracking (SCC) have been observed in underground reinforcement systems, which pose serious safety concerns for underground operations. A multitude of studies have focused on understanding the environmental factors, such as the composition of the corrosive medium, temperature, and humidity, in promoting the SCC of rock bolts, but the SCC failure mechanism associated with microstructural changes is still unclear due to the complexity of the underground environments. To understand its failure mechanism and develop effective mitigation strategies, this study evaluated different testing conditions, employing pin-loaded and bar-loaded coupon tests using representative specimens. The tests were conducted in an acidified sulfide solution. The failure characteristics and crack paths of the failed specimens were examined. It was observed that the steel with lower carbon content exhibited a reduced susceptibility to SCC. The subcritical cracks observed in the specimens were influenced by the microstructure of the material. SCC was observed not only on the original surface of rock bolts, which featured mill scale and decarburization, but also on freshly machined surfaces. Evidence for the occurrence of hydrogen-induced SCC was identified and discussed. The proposed testing methods and the obtained results contribute to a deeper understanding of SCC in rock bolts as well as promote the development of more durable materials for underground mining applications, ultimately enhancing the safety and reliability of rock bolt systems.

## 1. Introduction

In the excavation of underground resources and spaces, resin-grouted rock bolts play a pivotal role in maintaining effective ground control [1,2,3,4]. The high-strength bolts, typically measuring 22 mm in diameter and up to 2.4 m in length, feature helical rib patterns on the surfaces and are installed in pre-drilled holes in the mine roof, secured with polyester grout. However, in recent years, there has been a concerning surge in premature failures of the rock bolts in underground mines, primarily due to stress corrosion cracking (SCC) [5,6]. This phenomenon poses a significant threat to mine stability, operations, and personnel safety. SCC is an environmentally assisted cracking mechanism that adversely affects engineering materials, particularly metals [7,8,9]. It is a gradual process that occurs over time, driven by the synergistic interaction of corrosion and stress. The development of SCC necessitates three essential components: a susceptible material, specific chemical species present in the environment, and tensile stresses [10,11,12,13]. SCC progresses through distinct stages: crack initiation, crack propagation, and ultimate failure [14,15,16]. Generally, crack initiation occurs at surface flaws where stress concentrations are present, even at stress levels well below the macroscopic yield stress [17].

Given the complexity of SCC, its occurrence depends on the intricate interactions between materials and environments, leading to a variety of failure mechanisms [18,19,20]. As the industry strives for stronger and more durable steels, accurate testing becomes paramount. Testing conditions that could closely mimic real service scenarios would be necessary when assessing new materials or environments [21,22,23,24]. SCC failures are typically the result of prolonged tensile stress. To facilitate testing, coupons cut from actual components are often used instead of larger, more cumbersome parts [25,26]. Two widely adopted testing techniques are based on linear elastic fracture mechanics, employing pre-cracked specimens under static load, and slow strain rate testing, which applies a constant strain rate to smooth or pre-cracked specimens [27,28,29]. Standards for SCC testing have been established by organizations, for example, the American Society of Testing Materials (ASTM), with the aim to develop global standards to ensure consistency and reliability in testing procedures [30].

The susceptibility of alloys to SCC can be assessed through various testing approaches, each offering unique data on crack initiation, propagation, and failure times. Tension specimens, characterized by versatilities in type, size, and stressing methods, play a crucial role in determining tensile properties, generating SCC data, and investigating alloy–environment interactions [31]. Different specimen configurations, like notched tension specimens, provide valuable insights into specific failure mechanisms [32]. Surface preparation is a critical aspect, as improper handling can introduce adverse effects like hydrogen embrittlement [33]. In this study, experimental programs of replicating SCC failures of rock bolts in laboratory settings were conducted. Pin-loaded and bar-loaded coupons using representative alloyed steel specimens were immersed in corrosive solutions with different pH and sulfide concentrations. The effects of the different environmental factors as well as the SCC mechanisms in rock bolts were analyzed. This study contributes to a deep understanding of SCC in rock bolts as well as promotes the development of countermeasures for SCC, and ultimately enhances the safety and reliability of rock bolt systems in such environments.

## 2. Materials and Methods

### 2.1. Testing Specimens

To conduct the rock bolt SCC tests in the laboratory, the selection and preparation of appropriate specimens are important. For this study, two steel grades, including AISI 1355 (Type I) and 840 (Type II), were chosen due to their representative nature of the diverse steel compositions commonly utilized in manufacturing rock bolts for underground mining operations. Typically, rock bolts are constructed from a variety of steels, all exhibiting a ferrite/pearlite microstructure that ranges from approximately 85% pearlite in Type II to nearly 100% pearlite in Type I. Among the prevalent steel compositions used for rock bolt production, Type I (medium–high carbon) and Type II (medium carbon) are particularly illustrative, as they almost represent the full spectrum of microstructures encountered. To assess the hardness of the two types of rock bolt steel, a Struers Duramin-A300 hardness tester (Struers, Champigny sur Marne, France) was employed, with a load of 1 kg applied. Furthermore, a hardness traverse was conducted on each specimen using the same instrument but with a reduced load of 0.1 kg. For both hardness tests, a 606 HV standard was adopted. The chemical compositions of the steels and the mechanical properties of the specimens are detailed in Table 1.

**Figure 1:**
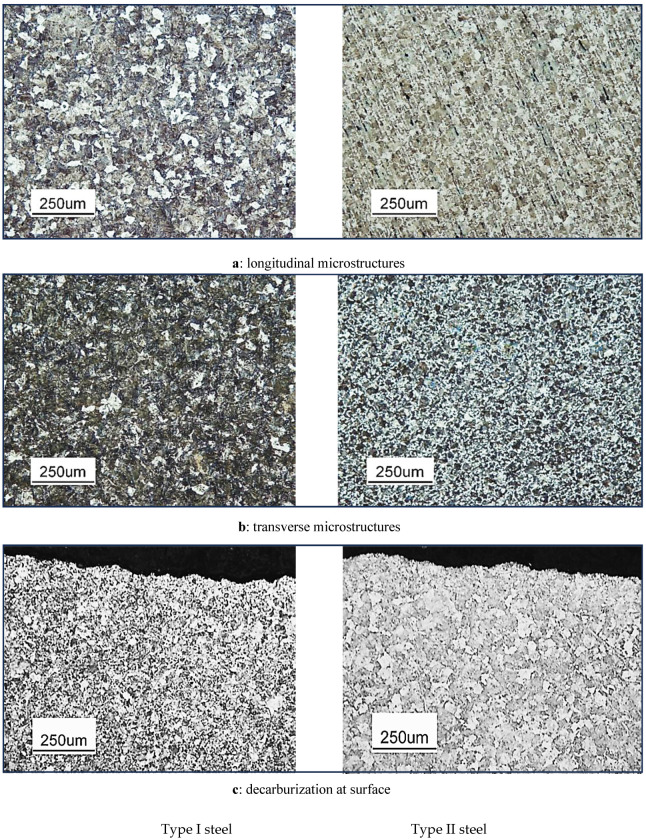
Optical micrographs of microstructure of Type I and Type II grade steels, (**a**): longitudinal and (**b**): transverse microstructures and (**c**): decarburization at surface.

The microstructures of steels play a crucial role in determining their mechanical properties and, consequently, their suitability for various applications [34,35]. A detailed examination of the longitudinal, transverse microstructures and decarburization at the surface of Type I and Type II steels are provided in Figure 1. Type I, also known as medium–high-carbon steel, exhibits a microstructure that primarily consists of pearlite and a smaller amount of ferrite (Figure 1a). The pearlite, which is a lamellar structure of ferrite and cementite, provides the steel with good strength and hardness. Type II, classified as medium-carbon steel, also has a microstructure dominated by pearlite, with some ferrite present (Figure 1b). However, the pearlite content and grain size may differ slightly from Type I due to variations in carbon content and processing conditions. The transverse microstructure also reveals grain boundaries more clearly, and the pearlite lamellae appear as alternating dark and light bands perpendicular to the direction of rolling or deformation. This perpendicular orientation gives the microstructure a distinct appearance compared to the longitudinal view. Decarburization refers to the loss of carbon from the steel’s surface during processing. Decarburization alters the microstructure of the affected area, typically resulting in a reduction in pearlite content and an increase in ferrite [36]. This change in microstructure can have a significant impact on the steel’s mechanical properties, particularly in the vicinity of the surface.

### 2.2. Stress and Corrosive Conditions

SCC, as an environmentally induced phenomenon and time-consuming processes, necessitates the consideration of testing environments for the development of accelerated SCC test methods. These methods are crucial for reliably predicting the SCC performance of alloys and elucidating the underlying mechanisms of SCC. Previous observations have highlighted the influence of mill scale and the underlying carburized layer on the surface of rock bolts, which alters their SCC behavior. To represent the full spectrum of microstructures, sections from rock bolts were selected as coupon specimens. Pin-loaded and bar-loaded coupons were used. Finite element analysis was utilized to comprehend the stress distribution within these specimens. In pin-loaded coupon specimens, the peak stress is concentrated near the inserted pin, representing the maximum applied stress. The bar-loaded coupon specimens experience modified stress conditions, with peak stresses occurring near the ends of the slot, particularly at the corners. The specimen designs and stress application methods constitute the foundation for a comprehensive exploration of SCC in rock bolts within a laboratory setting.

**Figure 2:**
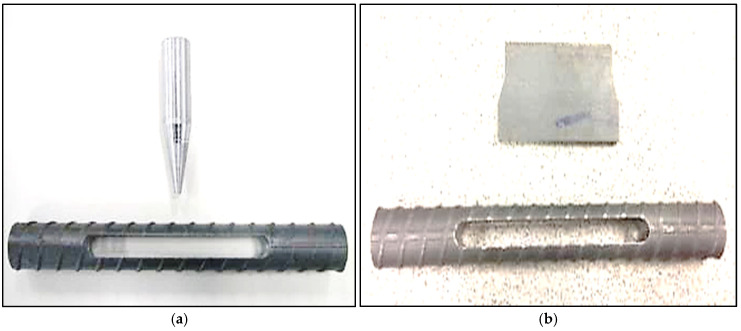
Slotted SCC rock bolt specimen (**a**): with tapered loading pin and (**b**): with a tapered loading bar.

For the preparation of the specimens, a Struers Discatom-6 water-cooled slitting machine was used to section the metallographic examination specimens perpendicular to the fracture surface. The specimens were ultrasonically cleaned in a water/detergent solution. To apply stress, a tapered pin or bar was inserted through a sectioned slot in the middle of the bolt specimen. The insertion of the pin induces plastic and elastic deformation around the slot, placing the test coupon under permanent stress—a crucial prerequisite for SCC to occur. Figure 2a depicts a typical coupon specimen with a loading pin, while Figure 2b showcases an alternative setup utilizing a loading bar. It is noteworthy that Type I and Type II steels possess ultimate tensile strengths that are 40% and 35% higher, respectively, than the yield stresses. This property enables a significant increase in stress in the coupon specimens above the yield stress, thereby accelerating the rate of SCC. The bar-loaded coupon specimen was employed to achieve stresses approaching 90–95% of the ultimate tensile stress, providing a more extreme testing condition compared to the pin-loaded variant. This setup facilitates in-depth insights into SCC behavior under high stress levels. For corrosive solutions, three test solutions were utilized, with their compositions based on an acidified 3.5% sodium chloride solution containing hydrogen sulfide, as previously reported to be reliable for simulating underground environments [37]. Adhering to standards is crucial for obtaining valid and accurate results. The solutions were specifically formulated to mimic the harsh and corrosive conditions that bolts experience in underground mining environments. To investigate the impacts of environmental factors, the loaded specimens were immersed in the corrosive solutions, which varied in pH and sulfide concentrations (Table 2).

### 2.3. Testing Procedures

For SCC tests, the coupon specimens were fully submerged in the test solutions, ensuring comprehensive exposure of all surfaces to the corrosive environment. This full immersion method was selected to maximize the interaction between the specimen and the test solution, mimicking the conditions where rock bolts are continuously immersed in mine water or other corrosive fluids. The time to failure of specimens under various testing conditions was recorded. A constant ambient temperature of 23 °C and a relative humidity of 50% were maintained. This controlled environment minimized the influence of external factors that could potentially interfere with the SCC process, enabling a more precise evaluation of how stress and the specific test solution interact to induce SCC. By carefully regulating these environmental parameters, this study was able to isolate the effects of the test solution on the SCC behavior of the rock bolts.

To observe the fractographic features of the specimens, examinations of fracture surfaces and the metallographic sections was carried out. After the tests, to facilitate the examination of the fracture surfaces, the test coupons were sectioned approximately 10 mm below the fracture surfaces using a Struers Discatom-6 water-cooled slitting machine. Prior to scanning electron microscope (SEM) examination, the fracture surfaces were initially inspected using a low-power stereo microscope. The fracture surfaces were first ultrasonically cleaned in a water and detergent mixture to remove any loose corrosion products. Subsequently, the specimens were ultrasonically cleaned in Ajax inhibited hydrochloric acid for a maximum of 10 s to eliminate the remaining corrosion products. Previous studies have demonstrated that ultrasonic cleaning in Ajax inhibited hydrochloric acid for up to 10 s effectively removes corrosion products from SCC failures in rock bolt steels without causing any significant damage to the fracture surface [38]. For the optical micrographs of the microstructure, the Olympus SZ40 low-power stereo microscope was used. The metallographic sections were examined using a Nikon 200 inverted optical microscope (Nikon, Tokyo, Japan). For the scanning electron microscopy (SEM) and electron backscattered diffraction (EBSD) images, a Nikon SEM operated at 15 kV with a working distance of approximately 10 mm was used. The specimens were examined uncoated and imaged using secondary electrons.

## 3. Results and Failure Characteristics

### 3.1. Testing Results

**Figure 3:**
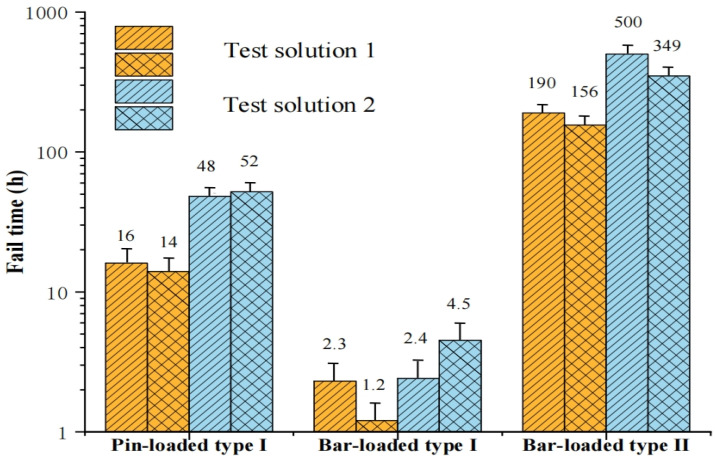
SCC tests results for specimens.

The results of the SCC tests are presented in Figure 3. The pin- or bar-loaded Type I specimens all failed within less than three days of exposure to the solutions. In contrast, all the pin-loaded Type II specimens exhibited no signs of cracking even after three months of immersion. This indicated that the Type II specimen were less susceptible to SCC under the specific solution conditions. While the pin-loaded Type II specimen did not fail, it did undergo SCC in the more highly stressed bar-loaded specimens. This observation could be attributed to the higher stress levels produced in these specimens, which further underscores the pivotal role of stress in accelerating the SCC process. For the bar-loaded specimens, the Type II specimens took substantially longer to fail compared to the Type I specimens. This finding indicated that Type II materials were less prone to SCC under the test conditions. The effects of pH and sulfide concentrations on SCC failure were also analyzed. For the pin-loaded Type I specimens, the specimen exposed to a higher concentration of sodium sulfide failed in approximately one-third of the time required for the specimen exposed to the less concentrated solution. Similar regulations were observed in the bar-loaded specimens. In contrast, the bar-loaded Type II specimen underwent failure in a lower concentration of sodium sulfide after 500 h, while failed in just 190 h in a more concentrated solution. This finding suggested that a more concentrated solution of sulfide generates hydrogen at a faster rate, thereby accelerating the SCC processes. This observation was consistent with a previous study that found that hydrogen plays critical roles in the occurrence of SCC [39]. Atomic hydrogen present in the environment can undergo reactions to form gaseous molecular hydrogen or diffuse into the bulk of the material as absorbed hydrogen. The rate of hydrogen absorption is primarily affected by surface adsorbates, which are commonly referred to as recombination poisons. Hydrogen sulfide is a common type of combination poison [37]. The recombination poisons facilitate hydrogen absorption by hindering the combination of atomic hydrogen to form hydrogen molecules. When the recombination of atomic hydrogen is delayed, the ability of atomic hydrogen to diffuse into the steel is enhanced.

### 3.2. Failure Characteristics

**Figure 4:**
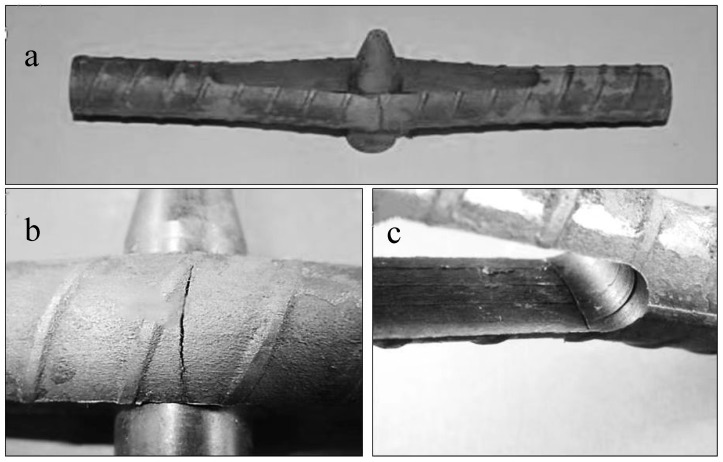
Failed pin-loaded specimens, (**a**): failed specimens, (**b**): crack at the pin, and (**c**): crack on the inside of the slot.

For the pin-loaded specimens, two typical failure modes were observed (Figure 4). The first failure mode involved cracking at the midpoint of the inserted pin (Figure 4b), where the finite element analysis model predicted the highest stress concentration. The second failure mode occurred inside of the slot at the corner of one of the ends (Figure 4c), where a second high-stress point was observed. All pin-loaded specimens exhibited cracking at both the midpoint of the inserted pin and the inside corners of the slot. In some cases, cracking occurred at both corners, while in others, it occurred at only one corner. Generally, the cracks originated from only one side of the bar, suggesting that the stress was slightly higher on one side compared to the other. This asymmetry was expected due to the necessary tolerance in the positioning of the slot, which results in slight differences in the material width on either side. Consequently, when the pin was inserted, one side of the specimen experienced higher stress, accelerating cracking on that side. Neither of the cracks propagated completely through the material. This was explained by the fact that each crack dissected the stress flow lines within the material. The deformation caused stress maxima at the inside edge of the specimen at the end of the slot and near the outside edge near the pin. As cracks propagated from both the inside and outside edges, they continued until they dissected the stress flow lines. Once this occurred, the cracks effectively relieved the stress, removing the essential stress component required for SCC propagation. Consequently, crack propagation ceased, preventing the cracks from fully penetrating the steel.

**Figure 5:**
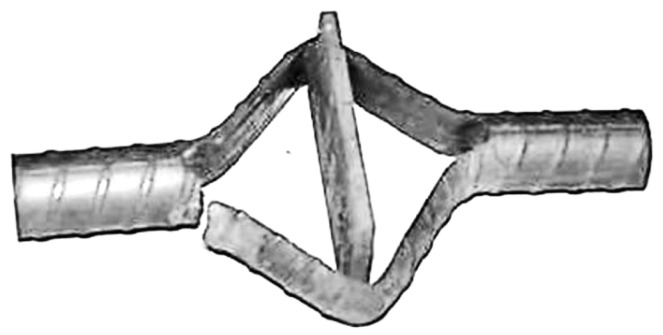
Failed bar-loaded coupon specimen.

The bar-loaded coupon specimens only exhibited one failure mode, originating from the corner at the end of the slot, and propagated through the thickness of the steel (Figure 5). The SCC cracks occurred on a particular side of the specimen. This phenomenon was attributed to the error associated with centering the slots cut in each specimen. Even a slight deviation from perfect centering was sufficient to cause non-uniform stress concentration, thereby promoting preferential SCC formation. It is important to note that the occurrence of a non-uniform stress distribution did not undermine the general validity of the test results. Rather, the observation of cracking behavior still provides valuable insights into the mechanisms of SCC in steel rock bolts. Unlike the pin-loaded specimens, the more highly loaded specimens failed exclusively from the inside corner, which was a machined surface. The machining processes often alters the shape, size, and surface finish of a material, and introduces small defects, stress concentrations, or changes in material properties. These factors can significantly influence the strength and durability of the specimen. Additionally, under high loads, the specimens undergo significant deformation. The deformation leads to the redistribution of stresses within the specimen, with tension and bend stresses becoming concentrated at the machined interior corners.

### 3.3. Crack Path Examinations

**Figure 6:**
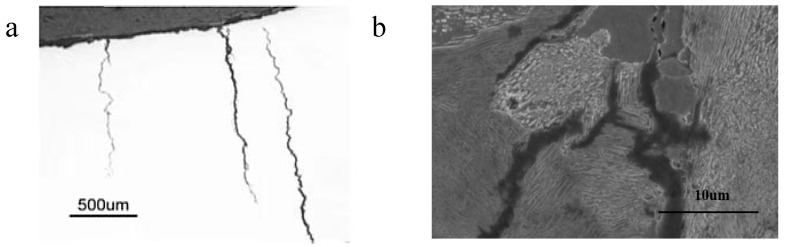
Observed cracks in rock bolt, (**a**): subcritical cracks and (**b**): crack propagated around pearlite colonies.

**Figure 7:**
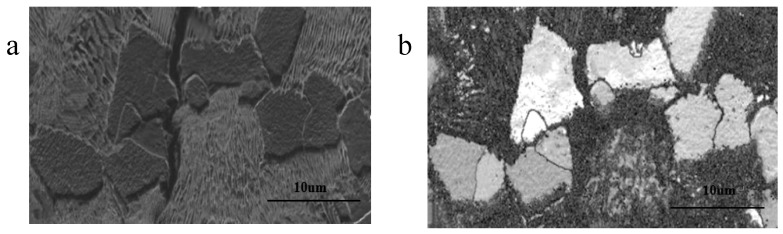
The crack propagated through the middle of a ferrite grain, (**a**): SEM images and (**b**): EBSD images.

The subcritical cracks were observed on the surface of the failed rock bolts with the aid of magnetic particle inspections. These cracks were narrow, with minimal corrosion evident on their walls (Figure 6a). The propagation behavior of these cracks was found to be influenced by the microstructural characteristics of the steel, particularly the arrangement and orientation of the pearlite and ferrite phases. The cracks exhibited a tendency to propagate around the pearlite colonies. When the lamellar structures within the pearlite were oriented roughly perpendicular to the overall direction of crack propagation (Figure 6b), the cracks tended to propagate along the boundaries separating adjacent pearlite colonies or along the interfaces between ferrite and pearlite. When the lamellae were oriented approximately parallel to the crack progression, the cracks tended to propagate between individual lamellae within the pearlite colonies. In contrast, the cracks propagated directly through the ferrite grains that lay in their path, bypassing them without significant deviation (Figure 7). This suggests that the ferrite grains, which are relatively stronger and more homogeneously structured, present a greater barrier to crack propagation compared to the weaker boundaries between pearlite colonies or between ferrite and pearlite interfaces. Similar observations have been documented in previous studies where SCC cracks propagated either through the ferrite/pearlite boundaries or along the pearlite lamellae, depending on the orientation of the pearlite lamellae relative to the direction of crack propagation [40].

The propagation behavior of cracks could be attributed to the microstructural arrangement and orientation of the pearlite and ferrite phases. The pearlite colonies in the steel consist of alternating layers of ferrite and cementite. When these lamellae are oriented perpendicular to the crack direction, the crack tends to propagate along the weaker boundaries between adjacent pearlite colonies or along the ferrite/pearlite interfaces, due to higher stress concentrations at these locations. In contrast, the relatively stronger and more homogeneous structure of the ferrite grains forces the crack to propagate directly through them when encountered [25]. Furthermore, the orientation of the pearlite lamellae relative to the crack direction plays a crucial role in determining the crack path. When the lamellae are oriented parallel to the crack progression, the crack is more likely to propagate between individual lamellae within the pearlite colonies, as the interfaces between lamellae may offered less resistance to crack propagation compared to the bulk material of the ferrite or pearlite phases.

## 4. Discussion

Test specimens subjected to higher strains exhibited more severe and accelerated rates of SCC, thereby confirming that the propensity for SCC increases with higher strains. This phenomenon was particularly evident in bar-loaded coupon specimens, which, due to the higher stresses they experienced relative to pin-loaded specimens, demonstrated more pronounced and rapid SCC progression. The corrosivity of the environment plays a pivotal role in determining the rate of SCC. Specimens immersed in solutions with higher concentrations of corrosive agents exhibited greater degrees of SCC compared to those exposed to solutions with lower concentrations. Corrosive agents in the environment attack the material’s surface, weakening it and thereby facilitating the initiation and propagation of cracks. Furthermore, Type II grade steel demonstrated increased resistance to SCC, with no failures observed in pin-loaded coupon specimens. In the case of bar-loaded coupon specimens, failure was observed within relatively short time periods. This indicated that, even in materials with high SCC resistance, extreme stress conditions could still lead to rapid crack propagation, highlighting the importance of considering both material properties and environmental factors in the assessment of SCC risk.

In the specimens where cracking occurred both from the outside and the inside corner, none of the cracks fully penetrated the section. In contrast, for specimens with a single crack, it extended through the entire section. Due to the stress-relieving effects, the crack on the inside corner disrupted the internal stress flow lines, reducing the stress on this side of the specimen. Consequently, the crack growing from the outside of the specimen eventually reached unstressed material at the cut flow line and ceased to grow. Gamboa and Atrens [41] conducted laboratory testing utilizing the linearly increasing stress test. They employed sulfate and chloride solutions with pH values of 2.1 and 1.8, and concluded that the stress required to induce failure was extremely high, approximating 95% of the ultimate tensile strength. In this study, pin-loaded specimens were loaded just above the yield strength, which was less than 70% of the ultimate tensile strength.

The surface of the bolt is covered with mill scale, which has been previously reported to elevate the risk of crack initiation. This is attributed to the mill scale significantly reducing the threshold stress of the surface, thereby providing a potential stress level within the cracking range [42]. In addition to the presence of mill scale, the surface of the rock bolts exhibits decarburization. Assessing the impact of decarburization on SCC is a complex task. While it may decrease the strength and increase the toughness of the material, which could potentially be beneficial for SCC resistance, it also alters the surface chemistry and microstructure. Consequently, the overall effect of decarburization on SCC may be either favorable or unfavorable, depending on the specific mechanisms involved. The ribs on the rock bolt surface are also locations of stress concentration, which are anticipated to affect SCC occurrence. While previous investigations based on tensile loading determined that rock bolts exhibited minimal influence of ribs on SCC occurrence [43,44], it is expected that under stress due to bending loading, the ribs will have a greater potential to influence local stress concentration.

The threshold for the occurrence of SCC in steels due to a hydrogen-induced mechanism is generally acknowledged to be at pH values of 4 or below [39]. Gamboa and Atrens [45] previously employed solutions with pH values within the range of 1.8 to 2.1 and put forward a mechanism for hydrogen-assisted SCC in rock bolt steels. Their hypothesis proposed that hydrogen, produced through cathodic reactions at low pH levels, diffused into the steel and accumulated in the triaxially stressed area preceding the crack tip. This accumulation resulted in the formation of a critical hydrogen concentration zone, which initiated the cracking process. As the crack advanced through this zone, it encountered material with insufficient hydrogen to sustain further cracking, leading to the cessation of the crack’s progression. Subsequently, hydrogen diffused into the subsequent triaxially stressed area ahead of the crack tip, and the process repeated. While the present study was limited by time constraints and could not conduct an exhaustive examination of the fracture surfaces, the observed characteristics were broadly in line with those reported by Gamboa and Atrens [45]. The test specimens displayed brittle cleavage in the overload region, further reinforcing the proposed hydrogen-induced SCC mechanism. They noted that the fractographic features of SCC in their specimens were virtually indistinguishable from those observed in service-induced SCC failures. Based on this observation, they deduced that the service failures were attributed to a hydrogen-induced mechanism. This finding may also be pertinent to rock bolt failures occurring in near-neutral pH mine waters, especially considering that pipeline steels have been known to fail through a hydrogen-induced mechanism in near-neutral pH groundwater [46].

**Figure 8:**
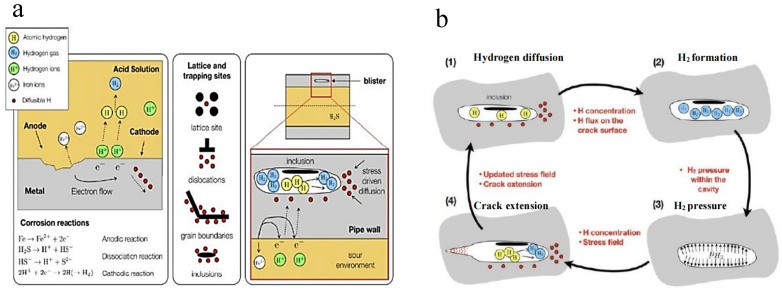
Hydrogen-induced cracking, (**a**): hydrogen accumulated at the interstitial sites and defects and (**b**): formation of hydrogen-induced crack.

The findings of the current study, in conjunction with the previous research conducted by Gamboa and Atrens [45], offer valuable insights into the mechanisms underlying stress corrosion cracking (SCC) in rock bolt steels. Based on extensive data derived from laboratory experiments, coupled with fractographic examinations of failed cable bolts, hydrogen-assisted SCC is proposed as the primary fracture mechanism. In corrosive underground mine environments, mining activities and strata movements result in the cracking of grout, thereby creating pathways for the diffusion of aggressive species into the bolts. This leads to the occurrence of general corrosion, which facilitates localized hydrolysis and the formation of localized corrosive environments at the base of corrosion pits [47]. Furthermore, the oxidation of chloride or sulfide minerals in underground water can cause a localized decrease in pH [48]. In acid environments, hydrogen emissions occur, and the generated atomic hydrogen diffuses into the metal lattice, accumulating at interstitial sites and defects within the metal structure (Figure 8a). The diffused hydrogen within the metal lattice weakens the bonds between metal atoms or combines to form molecular hydrogen, creating an internal stress field (Figure 8b). This internal stress field promotes the direct growth of cracks within the bolt, thereby accelerating the SCC failure process.

## 5. Conclusions

To understand the SCC failure mechanism and develop effective mitigation strategies, SCC rock bolt tests were conducted in environments that mimicked typical underground mining conditions, characterized by an acidified sulfide solution, using pin-loaded and bar-loaded coupon tests. The medium–high-carbon Type I steel with nearly 100% pearlite was more susceptible to SCC compared to medium-carbon Type II steel with 85% pearlite under the same solution conditions. For the pin-loaded specimens, two failure modes were observed, with one involving cracking at the midpoint of the inserted pin and the other involving cracking that occurred on the inside of the slot at the corner of one of the ends. The bar-loaded coupon specimens only exhibited one failure mode, originating from the corner at the end of the slot at the machined surface. Under high loads, the specimens undergo significant deformation, which leads to the redistribution and concentration of stresses at the machined midpoint and interior corners, promoting the initiation of SCC cracks. The dominant mechanism identified in this study was hydrogen-assisted SCC. The propagation of cracks was found to be influenced by the microstructural features of the steel, ultimately impacting the mechanical properties and durability of the rock bolts. A more concentrated solution of sulfide generates hydrogen at a faster rate, thereby accelerating the SCC processes. The proposed methods and obtained results enhance the understanding of SCC in rock bolts and can be used for the development of more durable materials and testing methods for underground mining applications.

## Figures and Tables

**Table 1:** Chemical compositions of rock bolt specimens.

Chemical Compositions (wt. %)												
Specimen	C	Mn	Si	Ni	Cr	Mo	S	P	Cu	V	Ti	Al
Type I	0.56	1.63	0.26	0.05	0.15	0.011	0.035	0.014	0.21	0.02	0.001	0.004
Type II	0.39	1.53	1.08	0.02	0.03	0.004	0.019	0.017	0.01	0.038	0.001	0.004
**Mechanical Properties**												
	**Yield (MPa)**		**UTS (MPa)**		**Elongation (%)**			**Impact Strength (Joules)**			**Hardness (HV 30)**	
Type I	600		850		12			9			326	
Type II	600		840		15			5			301	

**Table 2:** Test solution compositions.

Solution	Water (mL)	Sodium Chloride (g)	Acetic Acid (mL)	Sodium Sulfide (g)	pH
1	1940	60.0	60	3.0	3.4
2	1940	60.0	60	2.0	3.4

## Data Availability

The original contributions presented in this study are included in the article. Further inquiries can be directed to the corresponding author.
